# Crystal structure of TiBi_2_

**DOI:** 10.1107/S2056989016012391

**Published:** 2016-08-05

**Authors:** Kei Watanabe, Hisanori Yamane

**Affiliations:** a Institute of Multidisciplinary Research for Advanced Materials, Tohoku University, 2-1-1 Katahira, Aoba-ku, Sendai 980-8577, Japan

**Keywords:** crystal structure, CuMg_2_ structure type, titanium, bis­muth

## Abstract

Black granular single crystals of monotitanium dibismuth, TiBi_2_, were synthesized by slow cooling of a mixture of Bi and Ti from 693 K. The title compound is isostructural with CuMg_2_ (ortho­rhom­bic *Fddd* symmetry). Ti atoms are located in square anti­prisms of Bi atoms. The network of one type of Bi atom spirals along the *a*-axis direction while honeycomb layers of the other type of Bi atom spreading in the *ab* plane inter­lace one another.

## Abstract 2

Black granular single crystals of monotitanium dibismuth, TiBi_2_, were synthesized by slow cooling of a mixture of Bi and Ti from 693 K. The title compound is isostructural with CuMg_2_ (ortho­rhom­bic *Fddd* symmetry). Ti atoms are in the square anti­prisms of Bi atoms. The network of one type of Bi atom spirals along the *a*-axis direction while honeycomb layers of the other type of Bi atom spreading inn the *ab* plane inter­lace one another.

## Chemical context   

TiBi_2_ was first reported in the study of the Ti–Bi binary phase diagram by Vassilev (2006 ▸). Maruyama *et al.* (2013 ▸) confirmed the presence of TiBi_2_ in their phase-diagram study and showed that the powder X-ray diffraction (XRD) pattern was consistent with that of a Ti–Bi film prepared by RF sputtering (Simić & Marinković, 1990 ▸). However, the crystal system, lattice parameters and structure of TiBi_2_ were not reported.

In the present study, we prepared single crystals of TiBi_2_ to clarify the structure. The pellet of the starting mixture maintained the original shape after heating at 693 K. The powder XRD pattern of the sample showed that a mixture of TiBi_2_, Bi, and Ti had been obtained. Single crystals of TiBi_2_ approximately 120 µm in size were picked up from the fractured sample. TiBi_2_ is unstable and decomposes in air. When the mixture was heated at 703 K, the obtained sample was a mixture of Bi and Ti_8_Bi_9_. This temperature was above the peritectic temperature of TiBi_2_ (698 K) reported in the phase diagram by Maruyama *et al.* (2013 ▸).

## Structural commentary   

TiBi_2_ is isotypic with CuMg_2_ (Schubert & Anderko, 1951 ▸; Gingl *et al.*, 1993 ▸), NbSn_2_, VSn_2_, CrSn_2_ (Wölpl & Jeitschko, 1994 ▸; Larsson & Lidin, 1995 ▸), and IrIn_2_ (Zumdick *et al.*, 2000 ▸). TiSnSb is the only reported compound which contains Ti and crystallizes in the CuMg_2_-type structure (Malaman & Steinmetz, 1979 ▸; Dashjav & Kleinke, 2003 ▸). The crystal structure of TiSb_2_ adopts the CuAl_2_ type, while that of TiSn_2_ is not known. TiBi_2_ is the first binary compound that is composed of Ti and a group 15 element and has the CuMg_2_-type structure.

**Figure 1:**
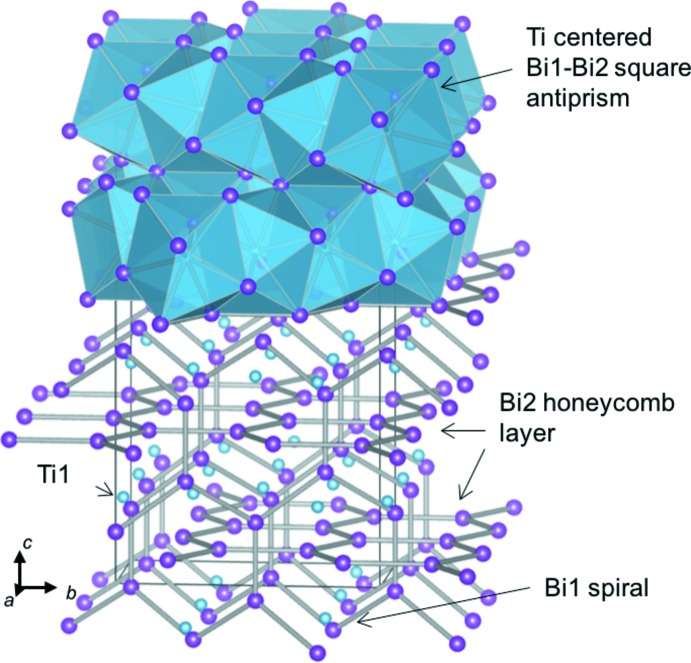
Crystal structure of TiBi_2_ illustrated with Ti-centered Bi1–Bi2 square anti­prisms and Bi1—Bi1 and Bi2—Bi2 bonds.

**Figure 2:**
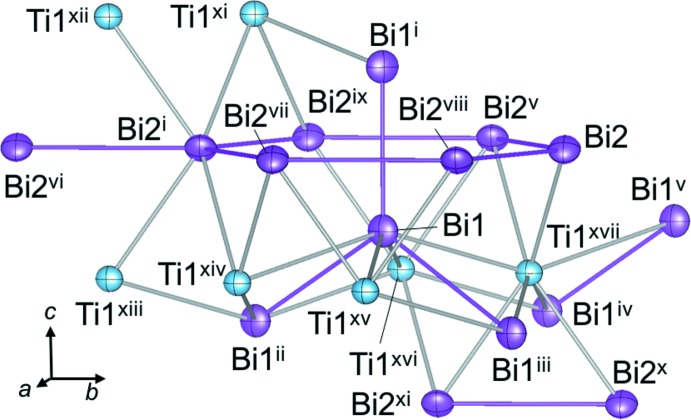
The atomic arrangement around Ti and Bi atoms in the structure of TiBi_2_. Displacement ellipsoids are drawn at 99% probability. [Symmetry codes: (i) *x*, −*y* + 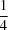, −*z* + 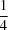; (ii) −*x*, −*y*, −*z*; (iii) *x* + 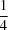, *y* + 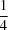, −*z*; (iv) *x* − 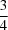, *y* + 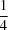, −*z*; (v) −*x* − 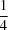, −*y* + 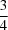, *z*; (vi) *x*, *y* − 1, *z*; (vii) −*x* + 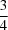, *y* − 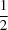, −*z* + 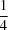; (viii) −*x* + 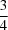, −*y* + 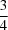, *z*; (ix) −*x* − 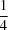, *y* − 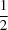, −*z* + 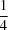; (*x*) −*x*, −*y* + 1, −*z*; (xi) −*x*, *y* − 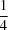, *z* − 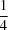; (xii) *x*, −*y* − 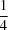, −*z* + 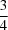; (xiii) *x*, *y* − 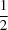, *z* − 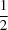; (xiv) *x* + 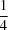, *y* − 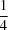, − *z* + 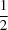; (xv) − *x* + 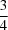, −*y* + 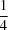, *z* − 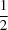; (xvi) −*x* − 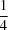, −*y* + 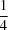, *z* − 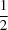; (xvii) −*x*, −*y* + 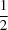, −*z* + 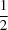.]

Fig. 1 ▸ shows the crystal structure of TiBi_2_ while the coord­ination environments of the Ti1, Bi1, and Bi2 atoms are illus­trated in Fig. 2 ▸. The Ti1 site is located in a square anti­prism of Bi atoms. The Bi square anti­prisms are aligned alternately along the *a* + *b* and *a* − *b* directions by sharing the square planes. Bi—Ti bond lengths in the Bi square anti­prism and the Ti—Ti distance of the inter-anti­prisms are 2.9382 (16)–3.0825 (6) and 2.9546 (2) Å, respectively, which are in the ranges reported for Ti_8_Bi_9_ [Bi—Ti = 2.818 (4)–3.144 (6) Å and Ti—Ti = 2.934 (6)–3.715 (5) Å; Richter & Jeitschko, 1997 ▸].

The Bi1—Bi1 bond lengths in the Bi1 spiral-like network are 3.0730 (8) Å in the *c*-axis direction and 3.4589 (4) Å in the other direction. The Bi2—Bi2 bond lengths in the Bi2 honeycomb layers in the *ab* plane are 3.4639 (8) Å in the *b*-axis direction and 3.3435 (4) Å in the other direction. The Bi—Bi bond lengths in the spiral rings and honeycomb layers in TiBi_2_ are in the range of those in Bi metal (3.071 and 3.529 Å; Cucka & Barrett, 1962 ▸). The inter­atomic distances between the Bi atoms of the spiral network and the honeycomb layers (Bi1—Bi2) are 3.6974 (3), 3.7309 (4) and 3.7546 (4) Å, which are longer than the Bi—Bi bond lengths in Bi metal.

## Synthesis and crystallization   

Starting powders of Bi (1 mmol, Mitsuwa Chemicals Co., Ltd, 99.999%) and Ti (0.5 mmol, Mitsuwa Chemicals Co., Ltd, 99.99%) were weighed, mixed in an alumina mortar with a pestle and formed into a pellet by uniaxial pressing in an Ar gas-filled glove box (O_2_ and H_2_O < 1 p.p.m.). The pellet was put in a tantalum boat (Nilaco Corp., 99.95%). The boat was sealed in a stainless-steel (SUS 316) tube. The sample was heated to 693 K in an electric furnace with a heating rate of 3.5 K min^−1^. This temperature was kept for 10 h, and then lowered to 473 K with a cooling rate of 5 K h^−1^. After cooling to room temperature by shutting off the electrical power to the furnace, the stainless-steel tube was cut and opened in the glove box. To identify the crystalline phases, powder XRD (Cu *K*α, Bruker, D2 phaser) was carried out for a portion of the sample which was ground in the alumina mortar and sealed under an Ar atmosphere in a holder with a kapton film window. The chemical compositions of TiBi_2_ single crystals placed on a carbon tape were determined with an electron probe microanalyzer (EPMA, JEOL, JXA-8200). Bi and TiO_2_ (Japan Electronics Co., Ltd) were used as standard samples. The analyzed composition ratio of Ti:Bi in the crystals was 1.0 (1):2.0 (1). A single crystal of TiBi_2_ was sealed in a glass capillary with Ar gas in the glove box for the single-crystal XRD experiment.

## Refinement   

Crystal data, data collection and structure refinement details are summarized in Table 1 ▸.

## Supplementary Material

Crystal structure: contains datablock(s) I. DOI: 10.1107/S2056989016012391/br2262sup1.cif

Structure factors: contains datablock(s) I. DOI: 10.1107/S2056989016012391/br2262Isup2.hkl

CCDC reference: 1497032

Additional supporting information: crystallographic information; 3D view; checkCIF report

## Figures and Tables

**Table 1:** Experimental details

Crystal data	
Chemical formula	TiBi_2_
*M*_r_	465.86
Crystal system, space group	Orthorhombic, *F* *d* *d* *d*
Temperature (K)	298
*a*, *b*, *c* (Å)	5.7654 (4), 10.3155 (6), 19.4879 (12)
*V* (Å^3^)	1159.00 (13)
*Z*	16
Radiation type	Mo *K*α
μ (mm^−1^)	123.50
Crystal size (mm)	0.14 × 0.09 × 0.06

Data collection	
Diffractometer	Bruker D8 goniometer
Absorption correction	Numerical (*SADABS*; Bruker, 2014 ▸)
*T*_min_, *T*_max_	0.016, 0.102
No. of measured, independent and observed [*I* > 2σ(*I*)] reflections	3881, 339, 309
*R*_int_	0.048
(sin θ/λ)_max_ (Å^−1^)	0.649

Refinement	
*R*[*F* ^2^ > 2σ(*F* ^2^)], *wR*(*F* ^2^), *S*	0.024, 0.062, 1.31
No. of reflections	339
No. of parameters	17
	
Δρ_max_, Δρ_min_ (e Å^−3^)	2.54, −3.80

Computer programs: *Instrument Service*, *APEX2* and *SAINT-Plus* (Bruker, 2014 ▸), *SHELXL2014* (Sheldrick, 2015 ▸) and *VESTA* (Momma & Izumi, 2011 ▸).

## supplementary crystallographic information

### Crystal data

**Table:**

TiBi_2_	*D*_x_ = 10.679 Mg m^−^^3^
*M**_r_* = 465.86	Mo *K*α radiation, λ = 0.71073 Å
Orthorhombic, *F**d**d**d*	Cell parameters from 4528 reflections
*a* = 5.7654 (4) Å	θ = 4.2–31.4°
*b* = 10.3155 (6) Å	µ = 123.50 mm^−^^1^
*c* = 19.4879 (12) Å	*T* = 298 K
*V* = 1159.00 (13) Å^3^	Granule, black
*Z* = 16	0.14 × 0.09 × 0.06 mm
*F*(000) = 3008	

### Data collection

**Table:**

Bruker D8 goniometer diffractometer	339 independent reflections
Radiation source: micro focus sealed tube	309 reflections with *I* > 2σ(*I*)
Detector resolution: 10.4167 pixels mm^-1^	*R*_int_ = 0.048
ω, φ scans	θ_max_ = 27.5°, θ_min_ = 4.2°
Absorption correction: numerical (SADABS; Bruker, 2014)	*h* = −7→7
*T*_min_ = 0.016, *T*_max_ = 0.102	*k* = −13→13
3881 measured reflections	*l* = −25→25

### Refinement

**Table:**

Refinement on *F*^2^	Primary atom site location: dual
Least-squares matrix: full	Secondary atom site location: difference Fourier map
*R*[*F*^2^ > 2σ(*F*^2^)] = 0.024	*w* = 1/[σ^2^(*F*_o_^2^) + (0.0275*P*)^2^ + 47.1241*P*] where *P* = (*F*_o_^2^ + 2*F*_c_^2^)/3
*wR*(*F*^2^) = 0.062	(Δ/σ)_max_ < 0.001
*S* = 1.31	Δρ_max_ = 2.54 e Å^−^^3^
339 reflections	Δρ_min_ = −3.80 e Å^−^^3^
17 parameters	Extinction correction: SHELXL2014 (Sheldrick, 2015), Fc^*^=kFc[1+0.001xFc^2^λ^3^/sin(2θ)]^-1/4^
0 restraints	Extinction coefficient: 0.00038 (3)

### Special details

**Table:**

Geometry. All esds (except the esd in the dihedral angle between two l.s. planes) are estimated using the full covariance matrix. The cell esds are taken into account individually in the estimation of esds in distances, angles and torsion angles; correlations between esds in cell parameters are only used when they are defined by crystal symmetry. An approximate (isotropic) treatment of cell esds is used for estimating esds involving l.s. planes.

### Fractional atomic coordinates and isotropic or equivalent isotropic displacement parameters (Å^2^)

**Table:**

	*x*	*y*	*z*	*U*_iso_*/*U*_eq_	
Bi1	0.1250	0.1250	0.04615 (2)	0.0064 (2)	
Bi2	0.1250	0.45710 (4)	0.1250	0.0066 (2)	
Ti1	0.1250	0.1250	0.49898 (10)	0.0050 (4)	

### Atomic displacement parameters (Å^2^)

**Table:**

	*U*^11^	*U*^22^	*U*^33^	*U*^12^	*U*^13^	*U*^23^
Bi1	0.0035 (3)	0.0076 (3)	0.0082 (3)	0.00176 (15)	0.000	0.000
Bi2	0.0058 (3)	0.0080 (3)	0.0060 (3)	0.000	−0.00212 (15)	0.000
Ti1	0.0039 (9)	0.0060 (9)	0.0051 (8)	0.0005 (8)	0.000	0.000

### Geometric parameters (Å, º)

**Table:**

Ti1—Bi2^i^	2.9382 (16)	Bi1—Bi2^xix^	3.7546 (4)
Ti1—Bi2^ii^	2.9382 (16)	Bi1—Bi2	3.7546 (4)
Ti1—Bi2^iii^	3.0051 (16)	Bi1—Ti1^xx^	3.0257 (6)
Ti1—Bi2^iv^	3.0051 (16)	Bi1—Ti1^xxi^	3.0257 (6)
Ti1—Bi1^v^	3.0257 (6)	Bi1—Ti1^vii^	3.0825 (6)
Ti1—Bi1^vi^	3.0257 (6)	Bi1—Ti1^i^	3.0825 (6)
Ti1—Bi1^vii^	3.0825 (6)	Bi1—Ti1^xxii^	4.9243 (16)
Ti1—Bi1^i^	3.0825 (6)	Bi1—Ti1^xxiii^	4.9243 (16)
Ti1—Ti1^viii^	2.9546 (2)	Bi1—Ti1^xxiv^	4.9348 (16)
Ti1—Ti1^ix^	2.9546 (2)	Bi1—Ti1^xxv^	4.9348 (16)
Bi1—Bi1^x^	3.0730 (8)	Bi1—Ti1^xxvi^	5.1110 (4)
Bi1—Bi1^xi^	3.4589 (4)	Bi2—Ti1^i^	2.9382 (16)
Bi1—Bi1^xii^	3.4589 (4)	Bi2—Ti1^xxiv^	2.9382 (16)
Bi2—Bi2^xiii^	3.3435 (4)	Bi2—Ti1^xxvii^	3.0051 (16)
Bi2—Bi2^xiv^	3.3435 (4)	Bi2—Ti1^xxviii^	3.0051 (16)
Bi2—Bi2^xv^	3.4639 (8)	Bi2—Bi1^xxix^	3.6974 (3)
Bi1—Bi2^xiv^	3.6974 (3)	Bi2—Bi1^xxx^	3.6974 (3)
Bi1—Bi2^xvi^	3.6974 (3)	Bi2—Bi1^xxxi^	3.6974 (3)
Bi1—Bi2^xvii^	3.6974 (3)	Bi2—Bi1^xxxii^	3.6974 (3)
Bi1—Bi2^xiii^	3.6974 (3)	Bi2—Bi1^xii^	3.7310 (4)
Bi1—Bi2^xviii^	3.7309 (4)	Ti1—Bi1^xxii^	4.9242 (16)
Bi1—Bi2^xii^	3.7309 (4)	Ti1—Bi1^xxiii^	4.9242 (16)
			
Ti1^i^—Bi2—Bi1^xxx^	100.12 (2)	Bi2^xiv^—Bi1—Ti1^xxiv^	36.352 (10)
Bi1^xxix^—Bi2—Bi1^xii^	100.174 (6)	Bi2—Bi1—Ti1^xxiv^	36.426 (10)
Bi2^xvi^—Bi1—Bi2^xviii^	100.175 (5)	Bi1^v^—Ti1—Bi1^xxii^	36.48 (2)
Bi1^xii^—Bi1—Ti1^xxii^	100.237 (12)	Bi1^x^—Bi1—Ti1^xxiv^	36.775 (14)
Bi1^xii^—Bi1—Bi2^xiii^	101.056 (6)	Bi2^xiv^—Bi1—Ti1^xxii^	37.501 (10)
Bi1^xi^—Bi1—Bi2^xvii^	101.056 (7)	Bi2^iii^—Ti1—Bi1^xxii^	48.51 (2)
Bi2^xvi^—Bi1—Bi2^xvii^	102.459 (10)	Bi1^xxix^—Bi2—Bi1^xxx^	49.109 (12)
Bi1^xxix^—Bi2—Bi1^xxxii^	102.460 (10)	Ti1^vii^—Bi1—Bi2^xix^	49.72 (3)
Bi1^xxx^—Bi2—Bi1^xxxi^	102.460 (11)	Ti1^xx^—Bi1—Bi2^xviii^	50.23 (3)
Bi2^xiii^—Bi2—Bi1^xii^	102.596 (8)	Ti1^vii^—Bi1—Bi2^xvii^	50.37 (3)
Bi2^xv^—Bi2—Bi1^xii^	103.120 (6)	Ti1^i^—Bi1—Bi2^xviii^	51.27 (3)
Ti1^xxi^—Bi1—Bi1^xi^	103.83 (2)	Ti1^xxi^—Bi1—Bi2^xiv^	51.93 (3)
Bi1^xii^—Bi1—Bi2^xvii^	103.916 (6)	Ti1^i^—Bi2—Bi1^xii^	52.331 (19)
Bi2^xiv^—Bi1—Bi2^xix^	104.912 (8)	Ti1^xxvii^—Bi2—Bi1^xxix^	52.441 (7)
Ti1^ix^—Ti1—Bi1^xxii^	105.95 (7)	Ti1^xxviii^—Bi2—Bi1^xii^	53.148 (19)
Ti1^xxiv^—Bi2—Bi2^xiii^	106.077 (14)	Bi2^xviii^—Bi1—Bi2^xii^	53.241 (9)
Ti1^i^—Bi1—Bi1^xi^	106.29 (3)	Bi2^xvi^—Bi1—Bi2^xix^	53.310 (6)
Bi1^x^—Bi1—Ti1^vii^	106.58 (4)	Ti1^xxiv^—Bi2—Bi1^xxix^	53.901 (6)
Ti1^i^—Bi2—Bi2^xv^	106.753 (12)	Ti1^vii^—Bi1—Bi1^xi^	54.737 (17)
Ti1^xxvii^—Bi2—Bi2^xiii^	106.976 (10)	Ti1^xxvii^—Bi2—Bi2^xv^	54.81 (2)
Ti1^xx^—Bi1—Bi1^x^	107.69 (4)	Ti1^i^—Bi2—Bi2^xiii^	55.32 (2)
Bi1^vi^—Ti1—Bi1^xxii^	108.14 (5)	Bi1^xxx^—Bi2—Bi1^xii^	55.502 (6)
Ti1^xxvii^—Bi2—Ti1^xxviii^	109.61 (4)	Bi2^xiv^—Bi1—Bi2^xvii^	55.865 (12)
Ti1^xx^—Bi1—Ti1^vii^	111.026 (7)	Ti1^xx^—Bi1—Bi1^xi^	56.289 (16)
Bi1^xi^—Bi1—Bi1^xii^	117.32 (2)	Ti1^xxi^—Bi1—Ti1^vii^	57.847 (3)
Ti1^ix^—Ti1—Bi1^v^	117.43 (3)	Ti1^viii^—Ti1—Bi2^iii^	59.07 (5)
Ti1^xxiv^—Bi2—Bi1^xii^	118.70 (2)	Ti1^xxiv^—Bi2—Ti1^xxvii^	59.609 (4)
Bi2^xiii^—Bi2—Bi2^xiv^	119.12 (2)	Ti1^ix^—Ti1—Bi1^vii^	60.113 (19)
Ti1^ix^—Ti1—Bi2^iii^	119.48 (9)	Bi2^ii^—Ti1—Ti1^viii^	61.32 (5)
Bi2^xvii^—Bi1—Ti1^xxvi^	119.656 (11)	Ti1^xxiii^—Bi1—Ti1^xxiv^	61.418 (6)
Bi2^xii^—Bi1—Ti1^xxii^	119.910 (14)	Bi1^xii^—Bi1—Bi2^xviii^	61.757 (12)
Bi2^i^—Ti1—Ti1^viii^	120.13 (9)	Ti1^viii^—Ti1—Bi1^v^	62.04 (2)
Ti1^i^—Bi1—Ti1^xxii^	120.34 (3)	Bi2^xv^—Bi2—Bi1^xxix^	62.067 (6)
Bi1^i^—Ti1—Bi1^xxii^	120.34 (3)	Bi2^xv^—Bi2—Bi1^xxx^	62.067 (6)
Ti1^viii^—Ti1—Bi1^vii^	120.39 (3)	Bi1^xi^—Bi1—Bi2^xix^	62.132 (6)
Bi2^ii^—Ti1—Bi2^iii^	120.391 (4)	Bi1^xii^—Bi1—Bi2^xiv^	62.742 (7)
Bi2^xiii^—Bi2—Bi2^xv^	120.438 (12)	Bi1^xi^—Bi1—Bi2^xviii^	62.826 (12)
Bi1^x^—Bi1—Bi1^xi^	121.338 (11)	Bi2^xiv^—Bi2—Bi1^xii^	63.380 (4)
Ti1^xx^—Bi1—Ti1^xxiv^	121.44 (3)	Bi2^xiv^—Bi2—Bi1^xxix^	64.221 (7)
Ti1^vii^—Bi1—Ti1^xxiv^	121.95 (3)	Bi2^xiii^—Bi2—Bi1^xxxi^	64.221 (7)
Bi2—Bi1—Ti1^xxvi^	122.061 (9)	Bi1^x^—Bi1—Bi2^xiv^	65.444 (6)
Bi1^vi^—Ti1—Bi1^vii^	122.154 (4)	Bi1^x^—Bi1—Bi2^xix^	65.843 (6)
Bi1^xxix^—Bi2—Bi1^xxxi^	124.135 (12)	Bi2^xix^—Bi1—Ti1^xxvi^	67.048 (12)
Bi2^xiv^—Bi1—Bi2^xviii^	124.499 (6)	Ti1^xxv^—Bi1—Ti1^xxvi^	68.10 (3)
Bi2^xviii^—Bi1—Bi2^xix^	124.958 (7)	Bi2^xviii^—Bi1—Ti1^xxvi^	68.52 (2)
Ti1^xxii^—Bi1—Ti1^xxvi^	129.41 (3)	Bi1^v^—Ti1—Bi1^vii^	68.975 (7)
Bi1^xii^—Bi1—Ti1^xxvi^	129.73 (2)	Ti1^xxiii^—Bi1—Ti1^xxvi^	69.162 (10)
Ti1^xxiv^—Bi1—Ti1^xxvi^	130.440 (11)	Bi2^i^—Ti1—Bi2^ii^	69.36 (4)
Bi2^xiv^—Bi1—Bi2^xvi^	130.889 (12)	Bi2^xiii^—Bi1—Ti1^xxvi^	69.407 (12)
Bi2^xix^—Bi1—Bi2	131.685 (13)	Bi2^xiii^—Bi1—Ti1^xxiv^	69.516 (7)
Bi1^xi^—Bi1—Ti1^xxii^	131.728 (9)	Bi2^iii^—Ti1—Bi2^iv^	70.39 (4)
Bi2^xviii^—Bi1—Ti1^xxiv^	135.259 (12)	Bi2^xix^—Bi1—Ti1^xxii^	70.622 (7)
Bi2^iii^—Ti1—Bi1^vii^	135.68 (5)	Bi2^xvii^—Bi1—Ti1^xxiv^	71.591 (8)
Bi2^i^—Ti1—Bi1^v^	135.83 (5)	Ti1^xxii^—Bi1—Ti1^xxiii^	71.66 (3)
Bi2^xii^—Bi1—Ti1^xxiv^	136.209 (11)	Bi1^xxii^—Ti1—Bi1^xxiii^	71.66 (3)
Ti1^xxi^—Bi1—Ti1^xxvi^	138.917 (11)	Ti1^xxi^—Bi1—Ti1^xxii^	71.85 (5)
Bi1^xxxii^—Bi2—Bi1^xii^	141.173 (9)	Ti1^viii^—Ti1—Bi1^xxii^	72.76 (6)
Bi2^xvii^—Bi1—Bi2^xviii^	141.174 (9)	Ti1^xxiv^—Bi1—Ti1^xxv^	73.55 (3)
Ti1^xx^—Bi1—Ti1^xxii^	143.52 (2)	Bi2^iv^—Ti1—Bi1^vii^	75.584 (18)
Bi1^v^—Ti1—Bi1^vi^	144.62 (7)	Bi2^iii^—Ti1—Bi1^v^	75.62 (3)
Ti1^xx^—Bi1—Ti1^xxi^	144.63 (7)	Bi2^i^—Ti1—Bi1^vii^	75.73 (3)
Ti1^i^—Bi2—Ti1^xxiv^	146.49 (2)	Bi2^ii^—Ti1—Bi1^vii^	77.12 (3)
Ti1^vii^—Bi1—Ti1^i^	146.84 (7)	Bi2^ii^—Ti1—Bi1^v^	77.437 (16)
Bi1^vii^—Ti1—Bi1^i^	146.84 (7)	Bi1^xii^—Bi1—Ti1^xxiv^	84.563 (17)
Ti1^i^—Bi2—Ti1^xxvii^	146.946 (8)	Ti1^vii^—Bi1—Ti1^xxvi^	85.661 (13)
Bi2^i^—Ti1—Bi2^iii^	146.946 (8)	Ti1^i^—Bi1—Ti1^xxiv^	85.87 (4)
Ti1^i^—Bi2—Bi1^xxix^	149.23 (2)	Ti1^vii^—Bi1—Ti1^xxii^	87.57 (2)
Ti1^xxvii^—Bi2—Bi1^xii^	149.475 (16)	Bi1^vii^—Ti1—Bi1^xxii^	87.57 (2)
Ti1^xx^—Bi1—Bi2^xiv^	150.350 (13)	Bi2^xiii^—Bi1—Bi2^xviii^	87.936 (5)
Ti1^i^—Bi1—Bi2^xvii^	151.051 (14)	Ti1^xxi^—Bi1—Ti1^xxiv^	88.00 (2)
Ti1^i^—Bi1—Bi2^xix^	151.659 (13)	Ti1^i^—Bi1—Ti1^xxvi^	88.706 (13)
Bi1^xi^—Bi1—Bi2^xiv^	152.907 (6)	Bi1^xxxi^—Bi2—Bi1^xii^	92.064 (5)
Bi1^xii^—Bi1—Bi2^xix^	153.267 (4)	Bi2^xii^—Bi1—Ti1^xxvi^	93.35 (2)
Bi1^x^—Bi1—Bi2^xviii^	153.380 (4)	Ti1^xxviii^—Bi2—Bi1^xxix^	93.992 (16)
Bi2^xiii^—Bi2—Bi1^xxix^	155.439 (6)	Ti1^i^—Bi1—Bi2^xiv^	95.236 (16)
Bi1^xi^—Bi1—Ti1^xxiv^	158.113 (19)	Ti1^xxiv^—Bi2—Bi1^xxx^	95.410 (14)
Bi2^xviii^—Bi1—Ti1^xxii^	161.981 (11)	Ti1^xxi^—Bi1—Bi2^xviii^	95.54 (4)
Bi2^i^—Ti1—Bi1^xxii^	162.533 (7)	Ti1^vii^—Bi1—Bi2^xviii^	96.63 (4)
Bi2^xiv^—Bi1—Ti1^xxvi^	165.35 (2)	Bi2^xvi^—Bi1—Ti1^xxii^	96.863 (15)
Ti1^viii^—Ti1—Ti1^ix^	178.47 (15)	Ti1^xx^—Bi1—Bi2^xix^	97.142 (14)
Ti1^xx^—Bi1—Ti1^xxvi^	30.870 (13)	Bi2^xvi^—Bi1—Ti1^xxiv^	98.027 (15)
Bi2^xvi^—Bi1—Ti1^xxvi^	34.47 (2)	Ti1^vii^—Bi1—Bi2^xiv^	98.391 (15)
Ti1^xxii^—Bi1—Ti1^xxiv^	34.877 (3)	Bi2^xix^—Bi1—Ti1^xxiv^	98.570 (15)
Bi1^xi^—Bi1—Ti1^xxvi^	35.089 (15)	Bi2^xii^—Bi1—Bi2^xix^	99.134 (9)
Bi1^x^—Bi1—Ti1^xxii^	35.832 (14)	Bi1^x^—Bi1—Ti1^xxvi^	99.91 (2)

Symmetry codes: (i) −*x*, −*y*+1/2, −*z*+1/2; (ii) *x*+1/4, *y*−1/4, −*z*+1/2; (iii) −*x*+1/4, −*y*+3/4, *z*+1/2; (iv) *x*, *y*−1/2, *z*+1/2; (v) *x*+1/2, *y*, *z*+1/2; (vi) *x*−1/2, *y*, *z*+1/2; (vii) −*x*+1/2, −*y*, −*z*+1/2; (viii) −*x*+1/2, −*y*+1/2, −*z*+1; (ix) −*x*, −*y*, −*z*+1; (x) −*x*+1/4, *y*, −*z*+1/4; (xi) −*x*, −*y*, −*z*; (xii) −*x*+1/2, −*y*+1/2, −*z*; (xiii) −*x*−1/4, −*y*+3/4, *z*; (xiv) −*x*+3/4, −*y*+3/4, *z*; (xv) −*x*+1/4, −*y*+5/4, *z*; (xvi) *x*−1/2, *y*−1/2, *z*; (xvii) *x*+1/2, *y*−1/2, *z*; (xviii) *x*−1/4, *y*−1/4, −*z*; (xix) −*x*+1/4, −*y*+1/4, *z*; (xx) *x*−1/2, *y*, *z*−1/2; (xxi) *x*+1/2, *y*, *z*−1/2; (xxii) −*x*+3/4, *y*, −*z*+3/4; (xxiii) −*x*−1/4, *y*, −*z*+3/4; (xxiv) *x*+1/4, −*y*+1/2, *z*−1/4; (xxv) *x*−1/4, −*y*, *z*−1/4; (xxvi) −*x*−1/2, −*y*, −*z*+1/2; (xxvii) −*x*+1/4, *y*+1/2, −*z*+3/4; (xxviii) *x*, *y*+1/2, *z*−1/2; (xxix) −*x*+3/4, *y*+1/2, −*z*+1/4; (xxx) *x*+1/2, *y*+1/2, *z*; (xxxi) *x*−1/2, *y*+1/2, *z*; (xxxii) −*x*−1/4, *y*+1/2, −*z*+1/4.
